# Rotation scarf + Akin osteotomy for severe hallux valgus with a new evaluation index: distance between the first and second metatarsals

**DOI:** 10.1186/s12891-022-05356-z

**Published:** 2022-05-05

**Authors:** Wenyong Xie, Hao Lu, Guicheng Li, Yusong Yuan, Hailin Xu

**Affiliations:** Department of Orthopedics and Trauma, Peking University People’s Hospital, Beijing, 100044 China

**Keywords:** Hallux valgus, Rotation scarf osteotomy, Akin osteotomy, Outcomes

## Abstract

**Background:**

Although rotation scarf + Akin osteotomy has been described for correcting hallux valgus deformity, the treatment efficacy of rotation scarf + Akin osteotomy for severe hallux valgus should be further studied. The purpose of our study was to evaluate the outcomes of rotation scarf + Akin osteotomy on severe hallux valgus.

**Methods:**

We conducted a retrospective study of patients with hallux valgus who underwent surgery using rotation scarf + Akin osteotomy in our hospital between June 2014 and January 2020. The parameters evaluated include (1) the hallux valgus angle (HVA), (2) intermetatarsal angle (IMA), (3) distal metatarsal articular angle (DMAA), (4) tibial sesamoid position (TSP), (5) the length of first metatarsal bone and (6) ratio between the vertical distance from the lateral of the first metatarsal head to the medial of the second metatarsal head and the vertical distance of lateral of the second metatarsal head to the medial of the third metatarsal head (MT-I to II/II to III distance). A visual analog scale (VAS) was used to evaluate the degree of pain before and at the last follow-up after the operation. The American Orthopaedic Foot & Ankle Society (AOFAS) Forefoot Score wasassessed before and at the last follow-up after the operation. Patient satisfaction assessment was also conducted at the time.

**Results:**

All radiological parameters including, HVA, IMA, DMAA and TSP,, significantly improved (*p* < 0.001). The length of the first metatarsal was shortened 3.1 mm on average. The MT-I to II/II to III distance was also reduced to 1.8 after surgery and 3.3 before surgery. The VAS score and AOFAS score was also statistically significant before operation and at the last follow-up after the operation (*p* < 0.001). Forty-one (82%) feet in patients were very satisfied or satisfied.

**Conclusion:**

Rotation scarf + Akin osteotomy is demonstrated to be safe, effective, and feasible for correcting severe hallux valgus. It can obtain good long-term correction with a low incidence of recurrence and metatarsalgia. Postoperative satisfaction and functional recovery of patients are significantly improved. The MT-I to II/II to III distance, a new evaluation indicator, can be better evaluate the correction of hallux valgus.

## Background

Hallux valgus deformity is due to medial deviation of the first metatarsal and lateral deviation of the great toe, with or without coexisting pronation and subluxation of the metatarsophalangeal joint, with a prevalence of 28.4% among adults [1]. It is a complex multifactorial cause with internal and external risk factors. It has a higher prevalence in women and increases with age [2]. It may cause pain, deformities, inability to fit certain shoes, ulceration over the medial eminence and even impair daily activities [3].

Surgical treatment is the optimal choice for moderate to severe hallux valgus deformity. It can relieve pain, correct the deformity, and improve function [4, 5]. More than 100 operative procedures were used for hallux valgus deformity treatment [5, 6]. Scarf osteotomy was extensively used and reviewed [7–9]. Severe hallux valgus was defined as ahallux valgus angle (HVA) ≥ 40° or IMA ≥ 20° and always accompanied with severe deformity and foot pain [10]. Scarf osteotomy has been getting a lot of attention since its appearance, but it is still controversial to treat severe hallux valgus with scarf osteotomy [7, 8, 11–13].

In 1992, Duke et al. [14] modified the surgical procedure method and introduced rotation scarf osteotomy. The osteotomy could maintain excellent stability and possibly greater correct the intermetatarsal angle (IMA), [11] which could decrease postoperative complications and recovery time [15]. Akin osteotomy is chosen by many doctors to correct phalanx deformities. Some authors regard the Akin osteotomy mandatory for hallux valgus correction [16, 17]. In recent years, rotation scarf + Akin osteotomy has become increasingly popular.

Although rotation scarf + Akin osteotomy has been described in some studies for correcting hallux valgus deformity, the treatment efficacy of rotation scarf + Akin osteotomy for severe hallux valgus should be further studied. The purpose of our study was to evaluate the outcomes of rotation scarf + Akin osteotomy for severe hallux valgus.

## Methods

This study was approved by the ethics review committee of our institution (2021PHB134-001). This is a retrospective review of a cohort of patients. All involved patients gave written informed consent for their clinical data to be used in this study. The patients all gave their written consent to have all their clinical data. All methods were carried out in accordance with relevant guidelines and regulations (eg. Helsinki declaration).

### Inclusion and exclusion criteria

We conducted a study of patients with hallux valgus who underwent surgery in our hospital between June 2014 and January 2020 by a single surgeon. The inclusion criteria for this study were (1) failed conservative treatment for 6 months, (2) hallux valgus angle (HVA) ≥ 40° or IMA ≥ 20° and (3) undergoing rotation scarf and Akin osteotomy. Patients with (1) ages < 18, (2) first metatarsophalangeal arthritis including rheumatoid arthritis, (3) failed previous hallux valgus surgery, (4) patients not suitable for surgical treatment and (5) psychiatric disorders that could not obtain incomplete follow-up data were excluded.

### Surgical technique

**Fig. 1:**
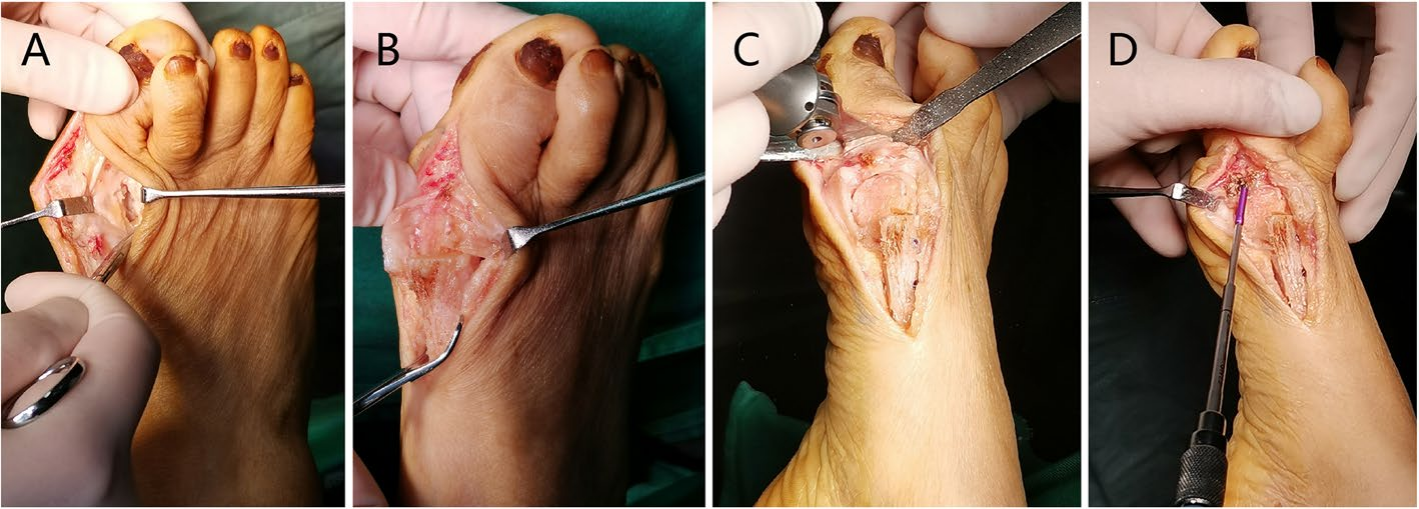
Intraoperative view of rotation scarf + Akin osteotomy. **A** Surgical view of lateral soft tissue release. **B** Scarf osteotomy after rotation and an adequate lateral movement. **C** The scarf osteotomy was fixed with two 2.0 cortical screws and Akin osteotomy was conducted. **D** The Akin osteotomy was fixed by one 2.0 cortical screw

Standard sterilization and draping techniques in the supine position with a tight pneumatic tourniquet were used for all patients underintraspinal or regional nerve anesthesia. And the regional nerves anesthesia was included femoral-sciatic nerve block or ankle block regional anaesthesia [18]. A 7 cm medial incision was made 1 cm distal to the first metatarsophalangeal joint over the hallux valgus and continuing proximally along the inferior margin of the first metatarsal, to the first metatarsocuneiform. A blunt dissection was conducted to avoid damage to the medial cutaneous nerve, and the joint capsule was incised longitudinally. In all patients, lateral soft-tissue release was performed through the medial incision through the dorsal skin, and the adductor hallucis tendon and lateral sesamoid ligament were released from their respective insertions in the metatarsal head and proximal phalanx (Fig. 1A).

Then, we resected the medial eminence of the first metatarsal at the sagittal groove. The long arm of the osteotomy line was conducted along the longitudinal axis of the metatarsal shaft, the greater the deformity was, the longer the osteotomy line was. Osteotomywas performed horizontally on both sides of the osteotomy line, and the entire osteotomy line was in a "Z" shape. The distal cut was on the dorsal aspect and the proximal cut was at approximately 45° angles from the medial proximal end to the lateral distal end to allow rotation to occur (Fig. 1B). Then, the lateral capsule was loosened, and the inferior fragment was rotated toward the second metatarsal to reduce the IMA. Our goal was to reduce the IMA into normal range. One millimeter of rotation was equal to one degree of correction [19]. Then two 2.0 headless compression screws were securely fixed. (Fig. 1C). All patients underwent Akin osteotomy of the proximal phalanx. Akin osteotomy was performed as a medial wedge and fixed with one 2.0 headless compression screw. (Fig. 1 C, D). Then the capsule was closed with 2–0 absorbable sutures, and the skin was closed with 4–0 silk sutures.

### Postoperative protocol

All patients completed the postoperative program. Wrap the hallux correction position with a crepe bandage. Two weeks after the operation, the suture was removed. The offloading orthosis in a rigid insole was used to carry weight 6 weeks later. After 12 weeks, patients were allowed to wear normal shoes and walk normally. It was allowed to return to sports after 6 months.

### Outcome measures

**Fig. 2:**
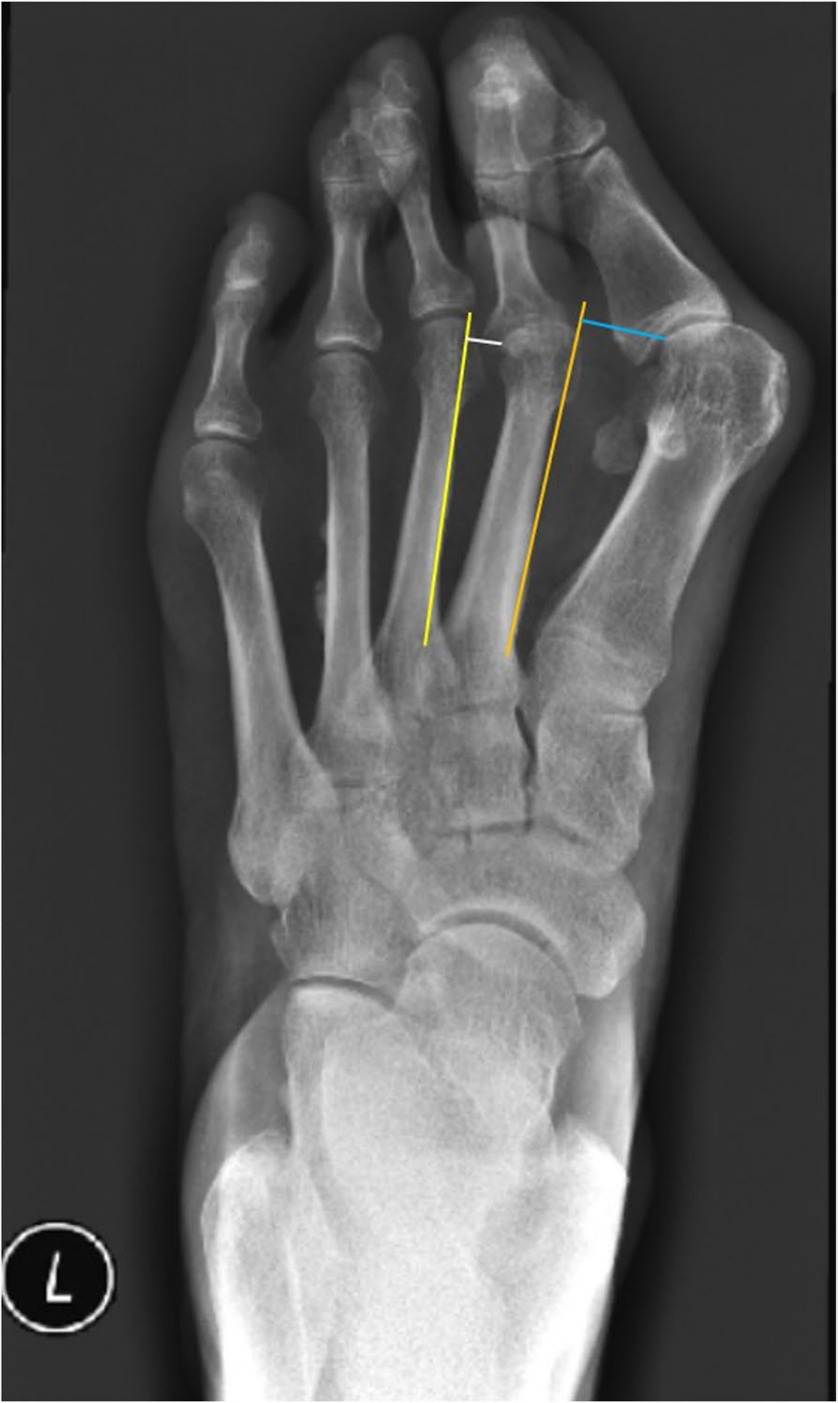
The measurement of distance between metatarsal heads in a severe hallux valgus. The orange line is parallel to the axis of first metatarsal and in the lateral of the second metatarsal head. The wathet-blue line is the distance from the lateral of the first metatarsal head to the medial of the second metatarsal head. The yellow line is parallel to the axis of second metatarsal and in the lateral of the third metatarsal head. The white line is the second metatarsal head to the medial of the third metatarsal head

Anteroposterior and lateral weightbearing X-ray were performed before and after the operation and the last follow-up, which can be used for the preoperative and postoperative measurements of radiographs parameters. Radiographs were analyzed in a digital manner using the Picture Archiving and Communication Systems (PACS). The parameters evaluated include (1) the HVA, (2) IMA, (3) distal metatarsal articular angle (DMAA), (4) tibial sesamoid position (TSP) in relation to the midshaft axis of the first metatarsal (7-part grading system) [20], (5) the length of first metatarsal bone and (6) ratio between the vertical distance from the lateral of the first metatarsal head to the medial of the second metatarsal head and the vertical distance of lateral of the second metatarsal head to the medial of the third metatarsal head (MT-I to II/II to III distance) As show in Fig. 2, the MT-I to II/II to III is the distance of wathet-blue line/the distance of white line. HVA is defined as the angle between the line from the center of the first metatarsal base to the center of the first metatarsal head and the line from the midpoint of the proximal articular surface of the proximal phalanx to midpoint of distal articular surfaces of the proximal phalanx [21]. The IMA is the angle of the line from the center of the first metatarsal base to the center of the first metatarsal head with the axis of the second metatarsal shaft [21]. DMAA is the angle between the vertical line of the distal articular surface of the first metatarsal and the line from the center of the first metatarsal base to the center of the first metatarsal head. The lengths of the first metatarsal and second metatarsal were measured absolutely by weightbearing X-ray. A visual analog scale (VAS) was used to evaluate the pain level before and at the last follow-up after the operation. The American Orthopaedic Foot & Ankle Society (AOFAS) Forefoot Score was assessed before and at the last follow-up after the operation. Patient satisfaction was analyzed though answering the question “Are you satisfied with the result of the surgery?” with 1 of 4 responses: (1) very satisfied, (2) satisfied, (3) undecided, or (4) not satisfied [22].

### Statistical analysis

Statistical analysis was performed using SPSS 23.0 (SPSS, Chicago, IL, USA). Continuous variables are described as median (interquartile range) or mean ± standard deviation for quantitative data and as numbers for categorical measures. Continuous normally distributed data were analyzed using the paired Student’s t test. Continuous nonnormally distributed paired data were analyzed using the Wilcoxon rank-sum test. *P* < 0.05 was considered significant.

## Results

A total of 42 patients with 50 feet met our inclusion criteria, including 29 on the right and 21 on the left. There were only 3 male patients. The average age at the time of surgery was 61 ± 9 years. Follow-up data were obtained in all 42 patients. The average follow-up time was 47.4 ± 20 months (Table 1).

**Table 1:** Patient demographics

Parameters	Value
Number of feet	50
Right feet (Number, %)	21(42)
Left feet (Number, %)	13(26)
Bilateral (Number, %)	16(32)
Age at time of surgery (years)	61 ± 9
Female (Number, %)	47 (94)
Male (Number, %)	3 (6)
Follow-up time(months)	47.4 ± 20

**Fig. 3:**
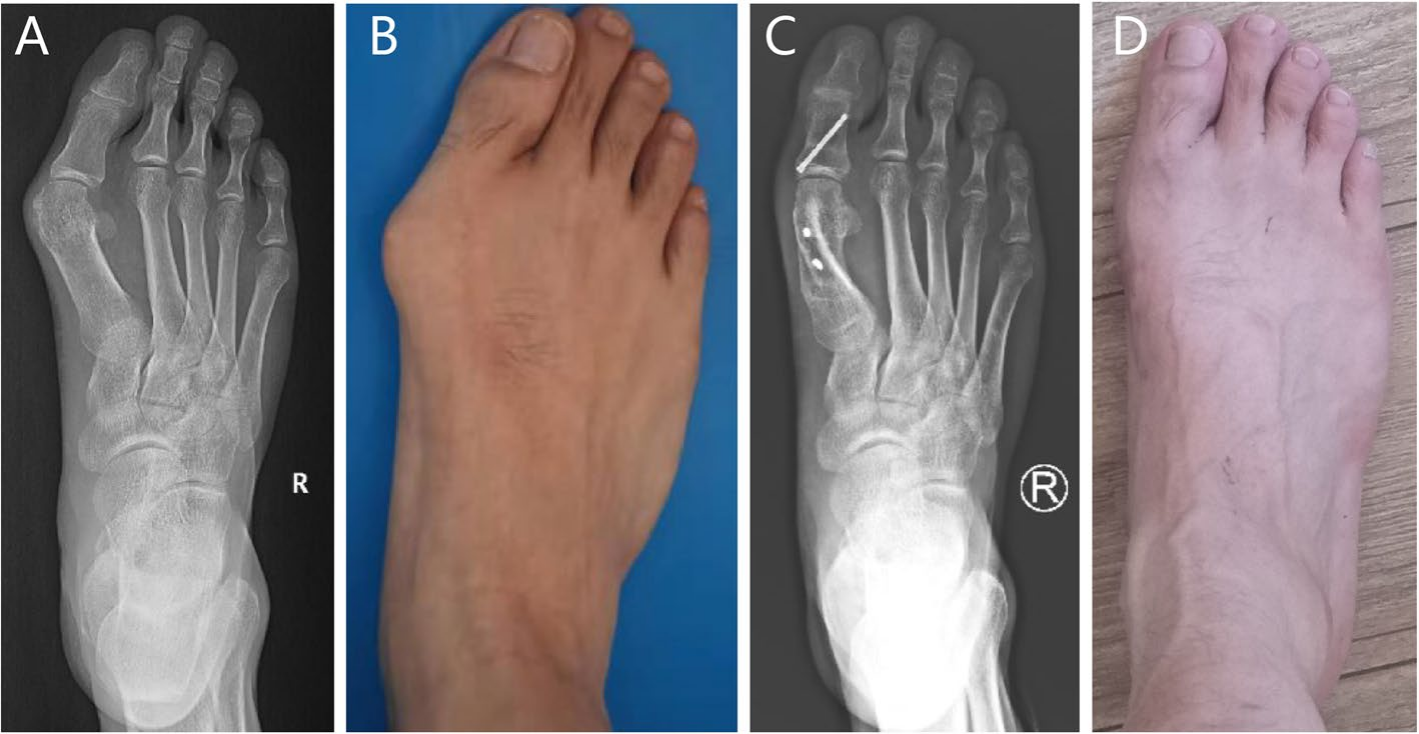
Preoperative (**A**, **B**) and follow-up (**C**, **D**) of a severe hallux valgus (HV) deformity corrected with rotation scarf + Akin osteotomy

The VAS score and AOFAS score before the operation and at the last follow-up after the operation were showed in Table 2 and the difference between the preoperative and postoperative stages was statistically significant (*P* < 0.001) (Fig. 3). All patients had substantial foot pain during daily life or sports activity before the operation.

**Table 2:** Preoperative and postoperative radiographic and clinical results

	**Preoperative**	**Postoperative**	**Mean correction**	***P* value**
HVA	45.7 ± 5.0	12.1 ± 7.2	33.6	< 0.001^a^
IMA	15.2 ± 3.4	4.2 ± 2.5	11	< 0.001^a^
DMAA	29.7 ± 11.8	16.0 ± 7.1	13.7	< 0.001^a^
TSP	6(5–6)	4(3–4)	2	< 0.001^b^
MT-I length	56.4 ± 4.4 mm	53.3 ± 4.5 mm	3.1 mm	< 0.001^a^
MT-I to II/II to III distance	3.3(2.9–3.9)	1.8(1.5–2.1)	1.5	< 0.001^b^
VAS	5(4–5)	0(0–1)	5	< 0.001^b^
AOFAS	35(32–40)	95(89–100)	60	> 0.001^b^

*IM* Intermetatarsal, *HV* Hallux valgus, *TSP* Tibial sesamoid position, *MT-I* First metatarsal bone, *MT-I to II/II to III distance* Ratio between the vertical distance from the lateral of the first metatarsal head to the medial of the second metatarsal head and the vertical distance of lateral of the second metatarsal head to the medial of the third metatarsal head, *VAS* Visual analog scale, *AOFAS* American Orthopaedic Foot and Ankle Society

^a^Values are given as the mean ± standard deviation, the statistical analysis was conducted using paired Student t test

^b^Values are given as the median (interquartile range), the statistical analysis was conducted using a Wilcoxon rank-sum test

**Fig. 4:**
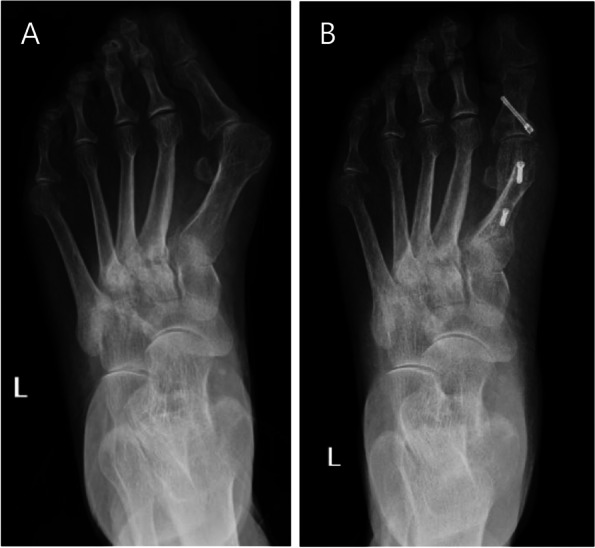
Preoperative dorsoplantar standing radiograph (**A**) and Follow-up dorsoplantar standing radiograph (**B**) of an extremely severe hallux valgus (HV) deformity corrected with scarf with Akin osteotomy showing the correction of the intermetatarsal angle, HV angle, distal metatarsal articular angle and tibial sesamoid position

The HVA decreased from 45.7° ± 5.0° before the operation to 12.1° ± 7.2° (*P* < 0.001) after the operation. The postoperative IMA reached 4.2° ± 2.5° from 15.2° ± 3.4° preoperatively (*P* < 0.001). The DMAA decreased from 29.7° ± 11.8° before the operation to 16.0° ± 7.1° (*P* < 0.001) after the operation, which was close to the normal range. Postoperative TSP reached 4 of 6 preoperatively (Table 2) (Fig. 4). The length of the first metatarsal was shortened 3.1 mm from 56.4 mm preoperatively to 53.3 mm postoperatively. The MT-I to II/II to III distance was also reduced to 1.8 after surgery with 3.3 before surgery (Table 2). Total 41(82%) feet in patients, including very satisfied (20 feet) and satisfied (21 feet), were satisfied with the treatment.

Complications occurred in 8 feet (16%). The recurrence of hallux valgus was defined as an HVA ≥ 20 [23]. Recurrence was found in 6 (12%) of the 50 feet at the time of the last follow-up. Among the 6 recurrent feet, one patient had superficial wound infections, which were treated with oral antibiotics and local wound care, and two patients experienced pain caused by transfer metatarsalgia. In the remaining 44 feet, one patient experienced pain caused by transfer metatarsalgia, and one patient developed hallux varus with metatarsal pain after the operation and was treated conservatively. The transfer metatarsalgia can be relieved by taking modified shoes or a brace. Table 3 showed the relationship between patient satisfaction and complications.

**Table 3:** Patient satisfaction

Patient satisfaction	Number (%)	Complication (Number)
Very satisfied	20(40)	Hallux varus (1), Recurrence (2)
Satisfied	21(42)	Recurrence (1)
undecided	6(12)	Recurrence with infections (1)
Not satisfied	3(6)	Recurrence with metatarsalgia (2) and Metatarsalgia (1)

## Discussion

Our study illustrates that rotation scarf + Akin osteotomy for severe hallux valgus was good in terms of treatment outcomes and patient satisfaction. Scarf osteotomy was conducted in the treatment of moderate and severe hallux valgus. However, clinical studies arerequired to further investigate its effects on severe hallux valgus. It was also indispensable for severe hallux valgus to conductsoft tissue release. In this study, all patients underwent rotation scarf + Akin osteotomy combined with soft tissue release, and the efficacy of the treatment was certain. The preoperative AOFAS score was 35, and patients had moderate to severe pain with significant deformity. The average age was 61, with a gender preponderance of females. Those were consistent with the other studies [9, 24].

Rotation scarf osteotomy had a better effect on the correction of IMA, and it also had a certain correction for DMAA [25]. Akin osteotomy combined with the first metatarsal osteotomy has been increasingly acknowledged [7, 9]. Through evaluating the treatment of severe hallux valgus with rotation scarf + Akin osteotomy we found that comparing to osteotomy alone or other proximal metatarsal osteotomy, rotation scarf + Akin osteotomy had many advantages, including the following: (1) Accurate correction of the IMA, one millimeter lateral displacement of the plantar head is equal to one degree of IMA correction, which can be evaluated and corrected intraoperatively [19]. (2) A large area of bone-to-bone contact reduces the risk of nonunion, and metatarsal shaft osteotomy reduces necrosis of the metatarsal head. (3) The high stability of the osteotomy, inferior and superior fragments of the metatarsal being pushed together by weight bearing forces rather than being distracted apart by those forces. (4) Rotation of the inferior fragment can also make the IMA greater correction. (5) Akin osteotomy can effectively correct the enlargement of DMAA, and effectively assist metatarsal osteotomy, which significantly improves the efficacy and satisfaction rate and fully compensates for the deficiencies exhibited by rotation scarf osteotomy [26]. Rotation scarf osteotomy was a Z-shaped diaphyseal osteotomy of the first metatarsal, which mainly completed deformity correction through translation and rotation [25]. During the operation, the length of the first metatarsal was maintained. The metatarsal head was displaced in a plantar direction as part of the rotation scarf osteotomy to reduce the possibility of transfer metatarsalgia. The first metatarsal is shortened after rotation scarf osteotomy, but the shortening is not very significant. Consistent with otherstudies, we believe that it has no effect on the prognosis [27]. Whether iatrogenic shortening of the first metatarsal contributes to transfer metatarsalgia is controversial, [28] but studies have shown that shortening up to 5 mm can lead to increased weight bearing on other metatarsal bones [29]. There were no additional complications in our study with a 3.1 mm shortening.

Common complications of rotation scarf osteotomy include first metatarsal fracture, transfer metatarsalgia, infection, medial dorsal cutaneous nerve injury of the hallux, and recurrence of deformity [8, 30, 31]. Among them, transfer metatarsalgia had a serious impact on patient satisfaction and three not satisfied patients all developed transfer metatarsalgia in our study. In our study, distal metatarsal osteotomies (Weil osteotomies) were conducted in the severe transfer metatarsalgia and the custom-made orthotics were used for mild transfer metatarsalgia. Smith et al. [31] reported that, based on early experience, there was a 6% perioperative complication for those beginning to master the scarf osteotomy. Before modifying this osteotomy, Coetzee et al. [8] reported a complication rate of 47%. In our study, it was found that complications were relatively rare, especially transfer metatarsalgia. The reason maybe that Weil osteotomy was conducted in the other metatarsal simultaneously with rotation scarf + Akin osteotomy when metatarsal had tenderness before operation and the shortening of first metatarsal was controlled well in rotation scarf osteotomy. The incidence of complications can be reduced when the surgeon isskilled in this osteotomy.

Hallux valgus deformity was often accompanied by enlarged DMAA. One of the reasons for recurrence deformity was that DMAA had not been well corrected [32, 33]. During rotation scarf osteotomy, DMAA can be corrected to a certain extent through pushing the inferior fragment lateral and rotating the inferior fragment. Then the match of metatarsophalangeal can be restored, and the rate of recurrence deformity can be reduced. In our study, DMAA decreased from 29.7° ± 11.8° before surgery to 16.0° ± 7.1° after surgery. It should be noted that although DMAA decreased after surgery, it did not return to a completely normal state. The DMAA can be reduced by changing the axis of the first metatarsal after rotation scarf osteotomy. The main effect of rotation scarf osteotomy is reflected in the recovery of HVA and IMA, and the corrective effect of DMAA is limited. On the other hand, rotation scarf + Akin osteotomy is also useful for the restoration of TSP which is associated with the prognosis of postoperative outcomes [23]. In our study, the effect for correction of HVA, IMA, DMAA and TSP by rotation scarf + Akin osteotomy were better than some minimally invasive and percutaneous techniques [34, 35]. Probably because the patients in our study is more severe in the deformation.

We propose a new evaluation indicator, MT-I to II/II to III distance, to measure and evaluate hallux valgus. We found that the vertical distance between the lateral first metatarsal head and the medial second metatarsal head increased with increasing severity of hallux valgus. In hallux valgus, the actual purpose of the osteotomy in the first metatarsal was to correct adduction of the first metatarsal. It was difficult to measure the HVA and IMA during and after surgery, and the DMAA also had variation after osteotomy in the first metatarsal. MT-I to II/II to III distance could also reflect the degree of hallux valgus, which was more accurate and convenient than HVA and IMA, especially during and after surgery. In our study, the MT-I to II/II to III distance was approximately 3.3 before surgery. We found that hallux valgus can be well treated and have pretty good outcomes when the vertical distance between the lateral first metatarsal head and the medial second metatarsal head was restored to 1.8 times the vertical distance between the lateral second metatarsal head to medial third metatarsals head. This method is more convenient to conduct intraoperatively.

Regarding the AOFAS and VAS scores, there was a significant difference before and after the operation. The postoperative AOFAS score increased by 60. The VAS score was also reduced to 0 in the follow-up time. Jeuken RM et al. [36] found even after a prolonged follow-up, scarf osteotomy scored approximately 32 points higher than preoperative measures in AOFAS. Perugia D et al. [9] studied scarf osteotomy for severe hallux valgus and the results were generally good and the mean correction was 54.1 in AOFAS. Biz C et al. [35] reported a pretty good result, which the mean correction AOFAS score was 65.3, in treatment of mild to severe hallux valgus by using Endolog device, an innovative titanium intromedullary nail. And a recent study about this technique (Minimally Invasive Intramedullary Nail Device) showed that the mean AOFAS score improved from 57.9 to 90.5 points in treatment of mild to severe hallux valgus [37]. A compare study between rotation scarf osteotomy and Endolog device can be conducted in the future. In patient satisfaction, we found that all three dissatisfied patients had transfer metatarsalgia, so there was a strong relationship between transfer metatarsalgia and dissatisfaction. Therefore, in the treatment of hallux valgus, we should pay attention to the occurrence of transfer metatarsalgia in addition to the correction of deformity.

Our study does have some limitations. First, the present study wasa retrospective without control group designed study. It cannot compare the advantages and disadvantages of rotation scarf + Akin osteotomy. Second, although the sample size of severe hallux valgus is relatively large, it is still necessary to increase the sample size for further investigation. Thirdly, the average follow-up time was 47.4 ± 20 months and there are patients who have a shorter follow-up time than others.

## Conclusions

In conclusion, rotation scarf + Akin osteotomy is demonstrated to be safe, effective, and feasible for correcting severe hallux valgus. It can obtain good long-term correction with a low incidence of recurrence and metatarsalgia. Postoperative satisfaction and functional recovery of patients are significantly improved. The MT-I to II/II to III distance, a new evaluation indicator, can be used to better evaluate the correction of hallux valgus. However, further studies with large sample sizes and long follow-up durations need to be performed to obtain more information about the long-term consequences of this surgical procedure.

## Data Availability

The datasets and materials are available from corresponding authors on reasonable request.

## Abbreviations

HVA: Hallux valgus angle
IMA: Intermetatarsal angle
DMAA: Distal metatarsal articular angle
TSP: Tibial sesamoid position
VAS: A visual analog scale
AOFAS: American Orthopaedic Foot & Ankle Society Forefoot Score
PACS: Picture Archiving and Communication Systems
